# Exploring the quality of life of school-aged children with disabilities in Saudi Arabia and their educational inclusion: from caregiver’s perspectives

**DOI:** 10.1186/s12889-025-25065-1

**Published:** 2025-10-31

**Authors:** Afnan Gmmash, Nourah Alamoudi, Shatha Alrehaili, Reem Basuodan, Mashael Alsobhi, Rawan Aldhabi, Abdullah Alqarni, Majed Albadi, Muataz Almaddah

**Affiliations:** 1 Department of Physical Therapy, Faculty of Medical Rehabilitation Sciences, King Abdulaziz University, P.O. Box 80324, Jeddah, 21589 Saudi Arabia; 2 Department of Rehabilitation Sciences, College of Health and Rehabilitation Sciences, Princess Nourah bint Abdulrahman University, P.O. Box 84428, Riyadh, 11671 Saudi Arabia

**Keywords:** Inclusion, Disability, Schools, Saudi arabia, Inaccessibility, Cross-sectional

## Abstract

**Background:**

Disabilities limit the children’s ability to use their body freely, fully engage with their surroundings, participate in the community, and access services. These restrictions could adversely impact their academic progress in schools. This study’s primary aim is to report the quality of life of school-aged children and the percentage of children attending schools. A secondary exploratory aim is to report factors related to educational inclusion.

**Methods:**

This study utilized a cross-sectional design using five domains from a validated quality of life tool targeting primary caregivers of children with disabilities. Data were analyzed using descriptive statistics and the Chi-square. 111 respondents met the inclusion criteria.

**Results:**

Regarding the children’s quality of life, less than half of the children feel happy about their interaction with children inside (35%) and outside of schools (39%). More than half of the children (51%) were happy with their interactions with their teachers, and in taking trips with their family (70%), as reported by their caregivers. In the participation domain, caregivers reported that children are happier when they participate in social outings. Most of the children were happy about utilizing their arms (53%), hands (51%), and drinking without assistance(53%). Caregivers stated that about 62% of the children were included in schools, and they were least happy with accessing special needs services in schools. This study also showed possible relationships between some demographic and quality of life factors with including children with disabilities in schools (*p* ≤ 0.05). Barriers that limit attending schools were reported.

**Conclusions:**

According to this study findings, the quality of life of children with disabilities is not ideal. Children face difficulties in interacting with their peers, participating in the community, using their body parts, and accessing services. This study provided preliminary data that suggest that not all children with disabilities are included in schools in Saudi Arabia. Although multiple factors were related to inclusion, they should be interpreted with caution. The reported difficulties could lead to unequal opportunities that interfere with the children’s quality of life and educational journey and should be considered to support the needs of children with disabilities.

## Background

According to the United Nations Children’s Fund, 150 million children under the age of eighteen years live with a disability [1]. The Saudi’s General Authority for Statistics classifies disability based on the Washington Group on Disability statistics to mobility, visual, communication, self-care, memory, and hearing disabilities [2]. According to recent figures, 1% of Saudi Arabia’s population has mobility-related disabilities, 0.6% have visual disability, 0.6% have communication disabilities, 0.5% have self-care related disabilities, 0.4% have memory loss related disabilities, and 0.3% have hearing disability, with the overall prevalence of disability hovering around 5.1%[3]. The number of persons with motor disabilities that lasted 0–19 years is 20,136 out of 187,892, which includes children as well as adults with motor disabilities [3]. In children, having a disability could limit meaningful participation in the community. For example, children with Down syndrome, cerebral palsy, autism, developmental coordination disorders, or traumatic brain injury have different types of disabilities, but their limitations affect their quality of life and educational progress [4, 5].

Disability is defined as any physical or intellectual disabilities that impacts a person’s ability to participate in any typical activities and social interactions in their environment. Enhancing the quality of life of children with disabilities using a holistic approach is crucial to promote their overall wellbeing. This includes boosting multiple aspects of their lives such as their physical, emotional and social development, and augmenting their ability to access a broad range of opportunities. Supporting these areas of development can be achieved by allowing the children to participate in educational settings, develop peer relationships, promote feelings of accomplishment and self-worth to assist them in living meaningful lives. Enriching the children’s quality of life is vital to improve their sense of autonomy and independence. One of the most important strategies to support the children’s quality of life is including them in educational settings with their typically developing peers [6, 7]. Educational inclusive practices refer to “teaching children and adolescents with disabilities in general educational settings besides their typically developing peers” [8]. Inclusive practices focus on educational quality rather than merely academic placement. Teachers, peer students, and the system all have a warm acceptance of children with disabilities and would like disabled children to make similar progress and growth as their typically developing classmates [9]. Since 1999 in Saudi Arabia, students with disabilities have been mainstreamed into schools, based on their type of disability [10]. While children with severe disabilities attend separate special education facilities, those with mild to moderate disabilities are incorporated into schools that provide a general education (GE) curriculum [11]. In 2008, the Saudi Arabian Ministry of Education established the structure of these programs [10]. In the United States, approximately 95% of children served under the Individuals with Disabilities Education Act attend regular schools. Children with developmental delays spent more time in general classrooms than children with intellectual or multiple disabilities [12]. Similar statistics are needed for every country to provide the necessary support for disabled children who are less frequently enrolled in regular classrooms. Children with disabilities’ quality of life is affected by a number of factors such as social participation, access to education, and physical as well as emotional health. Educational inclusion of children with disabilities can promote these factors by enabling daily interaction with typically developing peers and involvement in daily school activities. These activities play an important role in enhancing the children’s self-esteem, exposing them to various equal opportunities to achieve academic excellence, promoting their ability to participate in various physical activities, reducing the possibilities of discrimination, and promoting their acceptance in the community.

The focus of educational inclusive practices is on integrating disabled students in regular school settings. Inclusion means placing the needs and distinctive characteristics of each student first [13, 14]. One qualitative study explored the experiences of the employees in relation to including children with disabilities in one preschool in Riyadh city. The inclusion process had many positive effects. Some of these effects included enhanced psychological support for the children and their families, development of social and language competence, and fostering favourable beliefs toward people with disabilities [15]. Also, the study identified the following challenges faced in implementing the inclusion process: insufficient caregivers accompanying children with multiple disabilities in kindergartens, limitation in the transportation available for the facility; shortage of qualified educators, negative attitudes of others toward the children with disabilities, inacceptance of children with multiple disabilities, deficiency in the number of required services, and unsuitable school environment for children with multiple disabilities [15].

Children with disabilities are at a disadvantage when they start school and have lower persistence and progression rates than children without disabilities [16]. Recent statistics showed that 11% of Saudi adolescents with disabilities aged 10 to 19 years old are illiterate, 14% can only read and write, and 40% have completed primary school. Also, 17% have a university degree, and only 5.4% have diplomas [2].

The World Health Organization (WHO) describes the Quality of Life (QOL) as “a person’s perspective of their place in life, the culture in which they live, and in connection to their objectives, expectations, standards, and worries"[17]. The ability to participate in social activities is more restricted for those with physical disabilities than those without, which is linked to a lower degree of well-being, including a relatively worse quality of life [18].

Children with disabilities meet ongoing educational challenges. A study conducted in Kuwait found that barriers exist and could prevent children with disabilities from attending regular classrooms. These barriers could be imposed by teachers, social circumstances, academic requirements, and environmental or physical obstacles [19]. In addition, inclusion could be affected by the children’s type of disability [20]. Instructors in primary and secondary schools accept children with sensory and motor disabilities more than children with intellectual or emotional and behavioral difficulties [21]. People with motor disabilities suffer from maneuvering through restrooms, hallways, doorways, and rooms that are inaccessible or do not accommodate wheelchair users [16].

Including children in schools could result in improving the children’ participation in extracurricular activities, social gatherings, and family vacations [22]. In addition, engaging in formal education has several physical health benefits. School enrollment could also motivate the children to use their body more effectively and independently. Attending schools provide children with several possibilities to practice physical activities as well as socializing with their friends and the educational team [23]. The children’s type of disability could also play a factor in limiting the children’s quality of life [24]. There has not been much research conducted on exploring the quality of life of children with disabilities and their accessibility to education in Saudi Arabia. Indeed, the approximate number of children with disabilities enrolled in schools is not documented, and the barriers that could limit their enrollment have not been investigated. In addition, limited research explored the association between children’s participation and health outside of school with attending schools in Saudi Arabia. This study focuses on exploring the quality of life of children with disabilities in Saudi Arabia and provides an initial insight into possible demographic and quality of life factors associated with educational inclusion in Saudi Arabia.

## Methods

### Study design

This is a quantitative study with a cross-sectional design [25]. A cross-sectional design was used in this study to provide quantitative data related to the number of children with disabilities included in schools in Saudi Arabia. This research design also allowed to examine the correlation between the inclusion in schools with other factors. The authors used a self-developed survey using Google Forms that was then completed virtually by the parents or primary caregivers of children with disabilities. The study was conducted from August 2022 to June 2023.

### Study population

The participants were considered hard to reach communities; thus, they were recruited using convenience and snowball sampling strategies from different rehabilitation facilities and schools in Saudi Arabia. Eleven primary, secondary, and high schools were involved in the recruitment process. Also, social media was used to recruit study participants. Primary caregivers of children and teenagers with disabilities who met the inclusion criteria were invited to complete the survey. The children must be between 3 and 18 years of age, suffering from one or more of the following disabilities: visual disability, hearing disability, speech or language disability, autism, learning disabilities, emotional and behavioral disability, motor disability, and multiple disabilities. The participant must be the child’s primary caregivers. The primary caregivers should be able to read and comprehend either Arabic or English languages. Also, the participants and children must have lived in Saudi Arabia for at least one year in the last ten years to accurately reflect current practice. If the primary caregivers agreed to participate, a link containing the consent form and the survey questions was provided.

### Sampling

According to Saudi Arabia’s General Authority on Statistics, 187,892 people are disabled. 20,136 people are disabled with motor disability aged 0 to 19. G-power was utilized to estimate the sample size required to have a power of 80%. Using a medium effect size of 0.3 and a margin error of 0.05, the sample size required was 133.

### Procedure

The survey was developed based on the literature and previous similar studies. Online Google Forms were used to insert all the survey questions. This survey included a questionnaire that had been validated in Arabic [18]. The survey has five sections; the first section included a brief description of the purpose, length of the survey, the research team’s contact information, and the consent form. The next two sections included some exclusion and inclusion criteria related to the demographics of the children and their primary caregivers. The next section was related to measuring the quality of life, while the last section included two questions related to school inaccessibility that were retrieved from the parents’ survey in the Early Childhood Technical Assistance (ECTA) center [26].

### Instrumentation

The primary caregiver’s demographic questions included their age, gender, educational qualifications, relationship with the children, number of children with disabilities, order of the children among their siblings, and if he/she lives in Saudi Arabia and which region. The children’s demographic questions included children’s age, gender, educational inclusion (child enrollment/registration in school (yes or no question)), type of school (public, private, special education, or other), grade level, age of entering school, region of the school, type of disability, children diagnosis, and if the children and their caregivers have spent at least one year in Saudi Arabia in the previous ten years. There were three questions related to the geographic area, which included whether the participants live in Saudi Arabia, in which region, and where the school is located [2]. Personal data were not identifiable for the confidentiality and privacy of the participants.

Cerebral Palsy Quality of Life Questionnaire for Children (CP QOL-Child) Primary Caregiver Questionnaire (4–12 years) was used to measure quality of life. The scale was translated into Arabic 2016[27]. It is a 65-item questionnaire that should be completed by the primary caregiver. It includes questions that range from unhappy to happy. The included questions were the following: six items from the family and friends section, five from the participation section, six from the health section, three from the special equipment section, and three from the access to the services section. A total of 23 questions from the questionnaire were chosen for this study [28].

The tool was chosen to test the relationship between quality of life and inclusion. If children were included in the same environment as their typically developing peers, it could generalize to their actual lives. Including children in classrooms teaches them how to deal with their caregivers and the people around them, practice participation in the community, communicate with their peers and teachers, adjust their sleeping habits, and practice using special equipment in various settings. All of these areas can be examined by the cerebral palsy QOL-Child questionnaire [29]. This questionnaire is a reliable and valid tool used for children with cerebral palsy and has also been used on children with neurodisabilities [30] and on typically developing children [31]. This tool was translated to many languages, one of which is Arabic [27]. The Arabic as well as the English versions of this tool were used in our survey. This tool was utilized to determine the influence of QOL on the inclusion of children in school and with other variables.

According to Saudi Arabia’s Ministry of Health, disabled children can have any one of the following disabilities: visual disability, hearing disability, speech or language disability, autism, learning disabilities, emotional and behavioral disability, motor disability, and multiple disabilities. In this study, the authors used the Saudi Ministry’s classification in the survey to determine the child’s type of disability.

Lastly, the survey included two questions inquiring about the barriers faced by the primary caregivers when enrolling their children in school. Most common barriers based on the literature as well as an “others” options were provided for the participants to select from [32].

### IRB approval & consent forms

King Abdulaziz University, Faculty of Medicine’ Clinical Research Ethics Committee approved the study. A letter from the Planning and Development Department of the Ministry of Education was obtained to assist in distributing the questionnaire in schools. The consent form was included in the first section of the survey.

### Data analysis methods

Data were analyzed using the Statistical Package for the Social Sciences (SPSS) version 26. Frequencies and percentageswere used to anlayze the descriptive data. Chi-square analyses were performed to explore the association of the variables with inclusion. Statistical significance was detected at the level of 0.05.

## Results

A total of 185 participants completed the survey. We excluded 74 participants from the study who did not complete the survey or who did not have children with disabilities to avoid any unnecessary biases. A total of 111 primary caregivers completed the survey and were included in the analysis. Table 1 provides the demographic results of the primary caregiver and children. About 42.3% (*n* = 47) of the primary caregivers were between the ages of 37 and 47, and 72.1% (*n* = 80) were females. Most of the primary caregivers (61.3%, *n* = 68) have a bachelor’s degree. Some of the primary caregivers (10.2%, *n* = 11) stated having more than one child with a disability. Most of the participants (54.1%, *n* = 60) have male children, and 45.9% (*n* = 51) have female children with disabilities. Most children (31.4%, *n* = 33) were between 6 and 8 years of age. About 62% (*n* = 69) of the children were included in schools, most of whom started school at the age of six years (24%, *n* = 14), or seven years (20%, *n* = 12). Furthermore, most of the children (52%, *n* = 60) were males with disabilities, 60% (*n* = 36) of them were included in schools, and 40% (*n* = 24) were not included, as indicated by their primary caregiver. The majority of children (31.5%, *n* = 35) were in public schools. About 41.1% (*n* = 46) of all children have a physical disability, and 19.8% (*n* = 22) of the children had a confirmed cerebral palsy diagnosis. Most of the children with cerebral palsy (54%, *n* = 12) were included in the schools. Around 15% (*n* = 8) of the children with physical disability have no diagnosis. 60% (*n* = 25) of the children with physical disability were included in schools. Around half of the participants (61%, *n* = 68) were from the western areas, and 62% (*n* = 42) of them were included in schools. Most of the primary caregivers with a bachelor’s degree (66%, *n* = 45) had children who were included in schools.

**Table 1:** Demographics of the primary caregivers and children respectively

Variables	Frequency	Percentage
Age:		
Less than 18	19	17.1%
Between 18–25	6	5.4%
Between 26–36	33	29.7%
Between 37–47	47	42.3%
More than 47	6	5.4%
Gender:		
Female	80	72.1%
Male	31	27.9%
Relationship with children:		
Mother	88	79.3%
Father	20	18%
Sister	2	1.8%
Aunt	1	0.9%
Educational qualification:		
Primary school	6	5.4%
Middle school	6	5.4%
High school	20	18%
Bachelor	68	61.3%
Diploma	3	2.7%
Master	4	3.6%
PhD	2	1.8%
Less than primary school	2	1.8%
Children’s age:		
3–5	33	29.7%
6–8	35	31.5%
9–11	21	18.9%
12–14	12	10.8%
15–18	10	9%
Children’s Gender:		
Female	51	45.9%
Male	60	54.1%
Children registered in school:		
Yes	69	62.2%
No	42	37.8%
Type of school:		
Public	35	31.5%
Special education	14	12.6%
Private	14	12.6%
Day-care	4	3.6%
Currently not included in school	44	39.6%
Education level:		
Preschool	16	14.4%
Kindergarten	11	9.9%
Grade or year 1	11	9.9%
Grade or year 2	7	6.3%
Grade or year 3	3	2.7%
Grade or year 4	11	9.9%
Grade or year 5	2	1.8%
Grade or year 6	5	4.5%
Grade or year 7	3	2.7%
Grade or year 8	4	3.6%
Grade or year 9	1	0.9%
Grade or year 10	1	0.9%
Day-care	3	2.7%
Rehabilitation center	6	5.4%
Type of disability:		
Vision disability	5	4.5%
Motor disability	46	41.4%
Speech and language disability	2	1.8%
Emotional-behavioral disability	5	4.5
Learning disability	6	5.4%
Intellectual disability	2	1.8%
Multiple disability	45	40.5%
The children’s official diagnosis:		
Autism	5	4.5%
Spinal muscular atrophy	3	2.7%
Motor disability	18	16.2%
Vision disability	1	0.9%
Spina bifida	7	6.3%
Genetic	6	5.4%
Generally delayed growth	13	11.7%
Cerebral Palsy	23	20.7%
Down syndrome	9	8.1%
Optical atrophy	1	0.9%
Attention-Deficit Hyperactivity Disorder	2	1.8%
Cancer	3	2.7%
Multiple	3	2.7%
No diagnosis	17	15.3%

### Quality of life

Caregivers of children with disability ranked their children’s happiness on a scale from unhappy to extremely happy in five domains. In the family and friends domain, many of the families indicated that they are happy (happy to extremely happy) with how their children get along with other children in school (31.5%, *n* = 35), getting along with children outside of school (39%, *n* = 43), and in getting along with their teachers (52%, *n* = 58). 37% (*n* = 41) of the caregivers stated that from their point of view they think that their children feel happy with their ability to play on their own, with their friends (40%, *n* = 44), and in going out on trips with families (70%, *n* = 78). In the participation domain, the caregivers stated that their children feel happy about their ability to participate at preschool or school (38%, *n* = 42), sporting activities (*n* = 45,41%), in recreational activities (40%, *n* = 44), in social events outside of preschool or school (54%, *n* = 60), and in their community (41%, *n* = 45).

In the health domain, the caregivers documented that their children feel happy about the way they use their arms (53%, *n* = 59), the way they use their legs (39%, *n* = 43), and the way they use their hands (51%, *n* = 56). In the health domain, caregivers were also asked about how their children feel about their ability to complete daily activities. They were happy with their ability to dress themselves(37%, *n* = 41), drink independently (53%, *n* = 59), and use the toilet by themselves (26%, *n* = 32). Regarding the special equipment domain, the caregivers stated that their children were happy with the special equipment they have at home (28%, *n* = 31), at their school (23%, *n* = 25), and the special equipment available in the community (23%, *n* = 25). In the access to service domain, the caregivers reported that their children were happy with their access to community services and facilities (33%, *n* = 37), and their access to extra help with learning at preschool or school (25%, *n* = 28).

### Schools inaccessibility

**Fig. 1:**
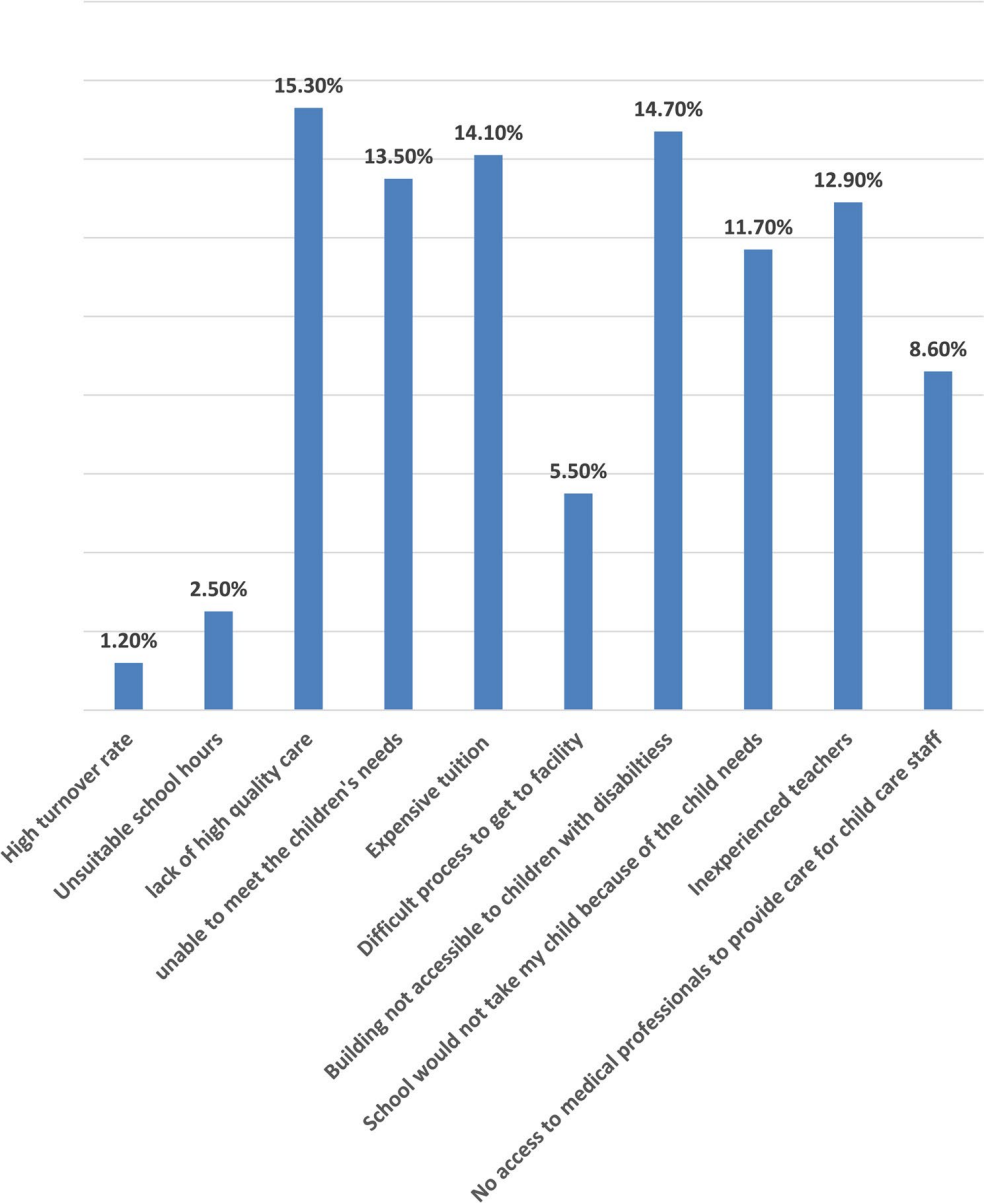
Describes the percentage of reasons that makes schools inaccessible

Figure 1 illustrates the percentage of reasons that limit the schools’ accessibility to children with disabilities.

### Factors associated with educational inclusion

Parental and child factors associated with educational inclusion are presented in Table 2. Quality of life domains associated with educational inclusion are presented in Table 3.

**Table 2:** Association between educational inclusion and the demographic variables

Variable		educational Inclusion		$\:{\varvec{x}}^{2}$	*p*-value
		Yes *n* (%)	No *n* (%)		
Children’s variables					
• Type of disability	Vision Motor Speech and language Learning Emotional-behavior Intelectual Multiple disability	3 (2.7) 27 (24.3) 1 (0.9) 5 (4.5) 4 (3.6) 2 (1.8) 27 (24.3)	2 (1.8) 19 (17.1) 1 (0.9) 1 (0.9) 1 (0.9) 0 18 (16.2)	136.129	< 0.001*
• Diagnosis	Autism Spinal muscular atrophy Motor disability Vision disability Spina bifida Genetic Generally delayed growth Cerebral Palsy Down syndrome Optical atrophy Attention-Deficit Hyperactivity Disorder Cancer Multiple No daignosis	4 (3.6) 1 (0.9) 12 (10.8) 1 (0.9) 3 (2.7) 2 (1.8) 8 (7.2) 13 (11.7) 6 (5.4) 0 2 (1.8) 3 (2.7) 2 (1.8) 12 (10.8)	1 (0.9) 2 (1.8) 6 (5.4) 0 4 (3.6) 4 (3.6) 5 (4.5) 10 (9) 3 (2.7) 1 (0.9) 0 0 1 (0.9) 5 (4.5)	122.854^a^	< 0.001*
• School’s geographical location	Central Northren Southeren Westeren Easteren Not registered	13 (11.7) 3 (2.7) 7 (6.3) 41 (36.9) 5 (4.5) 0	0 0 0 0 0 42 (37.8)	170.924	< 0.001*
• Child’s gender	Female Male	33 (29.7) 36 (32.4)	18 (16.2) 24 (21.6)	112.262	< 0.001*
• Child’s age (years)	3–5 6–8 9–11 12–14 15–18	15 (13.5) 19 (17.1) 18 (16.2) 10 (9) 7 (6.3)	18 (16.2) 16 (14.4) 3 (2.7) 2 (1.8) 3 (2.7)	16.827	0.010*
Parents’ variables					
• Parents age	Less than 18 Between 18–25 Between 26–36 Between 37–47 More than 47	11 (9.9) 3 (2.7) 20 (18) 31 (27.9) 4 (3.6)	8 (7.2) 3 (2.7) 13 (11.7) 16 (14.4) 2 (1.8)	114.926	< 0.001*
• Parents’ educational qualification	Primary school Middle school High school Bachelor Diploma Master PhD Less than primary school	5 (4.5) 2 (1.8) 10 (9) 45 (40.5) 2 (1.8) 2 (1.8) 2 (1.8) 1 (0.9)	1 (0.9) 4 (3.6) 10 (9) 23 (20.7) 1 (0.9) 2 (1.8) 0 1 (0.9)	120.812	< 0.001*
• Number of children	1 2 3 4 5 More than 5	9 (8.1) 14 (12.6) 21 (18.9) 11 (9.9) 9 (8.1) 5 (4.5)	8 (7.2) 9 (8.1) 12 (10.8) 7 (6.3) 5 (4.5) 1 (0.9)	1.06	0.302

*P < 0.05

**Table 3:** Association between educational inclusion and the children’s quality of life

QOL Domains		educational Inclusion		$\:{\varvec{x}}^{2}$	*p*-value
		Yes *n* (%)	No *n* (%)		
Family and friends	Unhappy Neutral Happy Unsure	8 (7.2) 14 (12.6) 31 (27.9) 16 (14.4)	3 (2.7) 11 (9.9) 19 (17.1) 9 (8.1)	6.803	0.558
Participation	Unhappy Neutral Happy Unsure	10 (9) 12 (10.8) 27 (24.3) 20 (18)	5 (4.5) 9 (8.1) 18 (16.2) 10 (9)	5.984	0.817
Health	Unhappy Neutral Happy Unsure	17 (15.3) 19 (17.1) 18 (16.2) 15 (13.5)	2 (1.8) 9 (8.1) 21 (18.9) 10 (9)	15.927	0.043*
Special equipment	Unhappy Neutral Happy Unsure	13 (11.7) 16 (14.4) 12 (10.8) 28 (25.2)	10 (9) 5 (4.5) 8 (7.2) 19 (17.1)	3.992	0.858
Access to services	Unhappy Neutral Happy Unsure	16 (14.4) 15 (13.5) 23 (20.7) 15 (13.5)	7 (6,3) 10 (9) 15 (13.5) 10 (9)	4.477	0.812

*P < 0.05

## Discussion

This study results suggest that the children with disabilities’ quality of life requires enhancement. Most of the caregivers reported that their children are not content with several areas related to their quality of life. In relation to educational inclusion, the majority of the caregivers reported that their children with disabilities are included in schools. However, limitations to this inclusion exist and may affect their successful participation in school. Also, this study’s findings proposed some statistically significant relationships that do not suggest causation and should be interpreted with caution. Some of these factors included the educational qualifications of the primary caregivers, children’s gender, children’s type of disability, quality of life, and geographic area.

The children’s interaction with their family and friends requires attention. It was reported in our study that the children were least happy with their interactions inside the school and in their ability to play independently. On the other hand, they were more delighted by outings with their families. This is consistent with what has been documented in other studies. Children with special needs have lower rate of meaningful peer interactions, and they are more disconnected from them [33]. Despite the challenges children with disabilities may face when participating in physical activities, studies show that children with disabilities enjoy informal outings. Schools should focus on involving children with disabilities in field trips and increasing their accessibility in these outings. In addition, more efforts should be dedicated to enhancing the interaction between children with and without disability [34, 35].

Our study suggested that children are not extremely happy with their participation in sports activities, recreational activities, and in their community. This is in line with the findings of others. Children with disability participate significantly less than their typically developing peers in the community [31]. Similarly, children with disabilities do not usually participate in sports activities. This is due to the inadequate environmental and community support. Similarly, in the access to services domain, children were unhappy with the provided access at school and in the community, as reported by their caregivers in our study. A study conducted in North America revealed that lack of adaptation and accessibility hindered children’s participation [36]. Educators should collaborate with caregivers to implement interventions that facilitate integrating children with disability in the community. Some of these interventions should be applied in schools by educating the typically developing children on ways to interact respectfully and support children with special needs in various settings. Schools should also create recreational programs that are tailored to support the needs of children with disabilities. Children spend half of their waking hours in schools; thus, fostering their abilities in schools is important to improve their overall quality of life [37].

In the health domain, children were least content with their ability to use the toilet, as suggested by their caregivers in our study. Additional training and involvement are required from the caregivers and educators to effectively toilet train children with disabilities [38]. Toilet training is a crucial skill that promotes independence, confidence, and development [39]. Therefore, individualized training programs should be employed for children with disabilities in collaboration with the educational staff. Numerous programs have been created and recommended in the literature that can be used to promote toilet training and enhance the quality of life of children with disabilities [40, 41].

Children in our study were unhappy with the available special equipment at home or in the community. A significant number of children with disabilities use special equipment to support their development and their accessibility within their community. Some of these assistive devices include walkers, orthotics, communication devices, standers, and seating devices [42]. All these devices require adjustments to meet the children’s special needs. Inadequate adjustments could lead to dissatisfaction with the use of these devices, which negatively impact the children’s confidence and wellbeing [43]. It is imperative to educate school professionals on the use of assistive devices for children with disabilities. The utilization of validated tools to assess the children’s satisfaction with the used equipment can play a major role in enhancing their ability to use them effectively and promoting their quality of life [44].

Our findings suggest that the primary caregiver’s educational qualification is significantly associated with the inclusion of children with disabilities in schools. This is not surprising as parents’ educational attainment has been significantly linked to children’s educational and behavioral outcomes [45]. Children of caregivers with lower educational qualifications were more likely to be inaccessible to schools due to their inability to meet their children’s needs. This was also evident in a study done in 2020 by a group of researchers as they found that the educational outcomes of children were highly associated with those of their parents [46]. More efforts should be dedicated to education and awareness to provide the necessary tools for parents to advocate for their children’s educational rights [47].

In this study, gender was significantly related to educational inclusion. According to the UNESCO report, females with disabilities face additional discrimination because of their gender [48]. It has been previously documented that disabled females face physical, social, and attitude impediments in comparison to disabled males or non-disabled female counterparts, which makes education a distant goal [48]. In addition, a study was done in India showed that one of the biggest obstacles for disabled females is frequently the architectural accessibility of school facilities, which include stairs, small hallways, inaccessible desks and equipment, and inaccessible bathrooms [49]. Sometimes, even typically developing females are more shy and fearful of judgments which could affect their overall participation [50]. This characteristic could be even more prominent in females with disabilities which could interfere with participation and hinder successful inclusion. In addition, a study reported that girls with mobility and other disabilities might be unable to use transportation networks, especially in poor nations, even if families allow their disabled daughters to attend a school away from home [48]. In Saudi Arabia, efforts are made to encourage equity as they adopt an anti-discrimination policy in which children have the right to free education and health services [51]. Children generally have a positive attitude toward their disabled peers in Saudi Arabia. However, collaboration between the educational team is warranted to implement inclusive practices successfully and eliminate any discrimination [52, 53].

Significant relationships between the type of disability as well as diagnosis with educational inclusion were evident in our study. Although poor school engagement is widespread among children with cerebral palsy [54], our results suggest that children with cerebral palsy and those with physical disabilities are more likely to have been included in schools compared to children with other types of disabilities. According to a study conducted in Australia, some children with cerebral palsy could have a mild disability that allows them to have greater public accessibility, and about 50% of them do not suffer from any intellectual disability [55]. These factors may have affected their inclusion. One Swedish study showed that several people with physical disabilities have proven to suffer from restrictions on participating in activities, and this significantly affects their quality of life [56]. On the other hand, in our study, 65.2% of all disabilities were physical disabilities, and most of the answers ranged from happy to very happy that their children’s quality of life was satisfactory. This could be related to their interactions with their non-disabled peers. Typically developing children in Saudi Arabian schools have more positive attitudes toward children with visible physical disabilities [52]. Mild to moderate disabilities receive general educational curricula in Saudi Arabia. However, it is not clear if they are more included in schools than children with other types of disabilities. This information must be clarified in future studies. In addition, researchers reported that policy makers must implement physical modifications of schools as well as adopt an Inclusive Special Needs Education (ISNE) program to improve the physically disabled children’s quality of life and education [57, 58].

A study conducted in Saudi Arabia found the most significant challenges associated with the inclusion process were the lack of qualified female teachers, negative attitudes of others toward people with disabilities, lack of acceptance of children with multiple disabilities, lack of services, and the absence of an appropriate school environment for children with multiple disabilities [15]. That qualitative study explored the experience of 14 participants recruited from one organization in one preschool in one city regarding integrating children with disabilities; a quantitative study on a larger sample was needed to add to their results. Thus, this questionnaire was published to all regions of the Kingdom. In this study results, barriers to inclusive education as reported by caregivers include the inability to find high-quality care, and experienced teachers to meet the children’s needs. This is consistent with what was found in other studies in Saudi Arabia and South Africa as barriers to children receiving an integrated education [57, 58]. One of these studies included the following as barriers to inclusive practices in schools: disorganized educational foundation, ineffective teachers, poorly teaching practices, and a society that is not yet ready to accept people with intellectual disability as members of society [59].

A study conducted in South Africa found that expensive tuition resulted in children being unable to afford the required resources, and teachers, parents, and even the government faced difficulties in providing and improving the education of those with disabilities [60]. Children with disabilities can receive free education from the Ministry of Education in the Kingdom of Saudi Arabia. Special education programs have been upgraded to help children with disabilities receive high-quality educational services in the least restrictive environment (LRE) [57]. The Regulation of Special Education Programs and Institutes of Saudi Arabia goal is to meet the educational needs of children with all types of disabilities. This regulation recommends early detection of disabilities and emphasizes the availability of free education for all children with disabilities [57].

This study found that the health domain from the quality of life questionnaire was significantly related to inclusion. That children’s ability to use their body parts and perform activities of daily living, such as toileting independently, could affect their school enrollment. This is expected as their ability to use their body parts, be self-sufficient, and manage toileting enable them to participate in the school environment effectively [61]. The provision of special adaptations and assessment of quality of life is recommended to improve the children’s success in school settings [62].

There were not enough studies to investigate the prevalence of inclusion based on regions in Saudi Arabia. The results of this study indicate that out of all regions of the Kingdom, the western region included most of the children with disabilities. However, more studies with bigger sample sizes are needed to compare inclusion with other regions. A clear description of the rights of disabled children in Saudi Arabia must be clarified in a family-friendly language for caregivers of children with disabilities. Some of the international examples include the early intervention services that are provided under the Individuals with Disabilities Education Act (IDEA) Part C to infants and toddlers, from birth to age 2, who have disabilities and their families, and special education and related services are provided to children and youth from the ages of 3 to 21 under IDEA Part B[63]. Both carefully describe the children’s inclusion rights and periodically publish statistics related to these acts. The Saudi government published similar statistics, but more focus on inclusion is encouraged. One of the main themes in Saudi’s 2030 vision promotes advances in education and health that is currently seen in many fields. Promoting inclusion in all of the education specialty areas can assist society in empowering children with disabilities and implementing the necessary medications that allow for successful inclusion and active participation of the minority into society. The Kingdom’s Ministry of Education website provides an elaborate Arabic description of the equality in education in Saudi Arabia. They also provide important links for educational facilities for children with disabilities [64]. Increasing the public’s awareness about these resources is necessary to promote inclusion.

To promote the success of children with disabilities in schools, therapy services must be available in school systems [65]. An example is the provision of pediatric physical therapists who can educate students, primary caregivers, and teachers on strategies to assist children in overcoming their physical limitations [66]. The American Physical Therapy Association (APTA) defines “best practice” as services that “assist children with disability to benefit from special education; therefore, physical therapists should primarily work in the classroom or elsewhere in the school/community that supports the student’s special education program.“[67]. Other services, such as speech therapy and occupational therapy, can promote the students’ ability to communicate and achieve superior academic achievements [68].

### Limitations

This study had some limitations. The authors used a validated tool for cerebral palsy on non-cerebral palsy children. Ideally, a validated questionnaire on children with all types of disabilities should be used. However, many assessment tools were initially validated on children with cerebral palsy and later on children with other disabilities [69]. In addition, this tool has been used on children with other disabilities. The authors did not reach the required sample size due to the unavailability of the contact information of the primary caregivers of children with disabilities in schools. Similar sample sizes were obtained from similar studies conducted on a similar population. A study was conducted in Saudi Arabia on the obstacles to providing special education services to students with attention-deficit/hyperactivity disorder in the Kingdom of Saudi Arabia, and the study sample consisted of (*n* = 96) individuals working in twenty-four governmental primary schools [59]. Our study included only certain factors associated with inclusion, but other factors that may contribute to inclusion, such as parents’ income, were not investigated. Not all barriers that affect educational inclusion were investigated in this study. While the used analysis in this study revealed an association between variables, causation cannot be inferred from this type of analysis.

### Recommendations for future studies

Future research must focus on other factors that have not been studied to improve the inclusion of children with disabilities in schools. This study provides important baseline data in which other researchers could expand. Other studies should analyze the relationship between the presented variables while controlling for any confounding variables. Barriers to inclusion must be investigated more extensively from the educators, policy makers, as well as older children’s perspectives. All teachers, school therapists, parents, and other family members must be willing to work together to support the children’s quality of life. Investigating successful collaboration with educators, experts, parents, and peers is promoted to enhance the integration of children with disabilities into socity [59].

## Conclusion

The quality of life of children with disabilities is less than optimal. Children with disabilities struggle to interact with their surroundings and participate in their community. Caregivers stated that not all children with disabilities are enrolled in schools, which could be due to the high tuition rates and lack of high quality care required to address the children’s special needs. More efforts should be directed toward improving the children’s quality of life by making the community and educational services more accessible to children with disabilities.

## Data Availability

The datasets used and/or analyzed during the current study are available from the corresponding author on reasonable request.
